# Schroth treatment results in AIS at high risk for surgery - 2 case studies

**DOI:** 10.1186/1748-7161-9-S1-O48

**Published:** 2014-12-04

**Authors:** Lior Neuhaus Sulam, Iris Braz

**Affiliations:** 1 Israeli Scoliosis Center, Tal Aviv, Israel

## 

Scoliosis is a 3D deformity of the spine combined with pathological structural changes.

Scoliosis tend to progress through growth.

BSPTS method based on Schroth principles is a specific physiotherapeutic treatment method for scoliosis. It considers the 3d changes of the spine, the geometrical and axial torsion of the hole spine, thorax and pelvic. Using specific exercise to achieve better muscle balance and corrected posture in order to prevent progression, improve esthetic and function.

## Aims

To present the efficacy of the BSPTS method based on Schroth principles treating progressive moderate AIS in 2 case studies using objective measures.

## Case 1

Starting point- age 17.5 years, Risser=2+, Rt thoracolumbar L3-T7 52° cobb, Trace 10/11, scoliometer 16° rt hump, plumb line 2.5 cm rt from center of sacrum.

End point- age 19, Risser = 4, Rt thoracolumbar L3-T7 35° cobb, Trace 6/11, scoliometer 12° rt hump, plumb line 1 cm rt from center of sacrum.

## Case 2

Starting point- age 14.5 years, Risser=1, Rt Tx 53° cobb, Trace 9/11, scoliometer 17° rt hump, plumb line 2 cm rt from center of sacrum.

End point- age 15.9, Risser = 3 Rt Tx 30° cobb, Trace 5/11, scoliometer 9° rt hump, plumb line 1 cm rt from center of sacrum.

## Conclusions

In spite of the late diagnosed moderate AIS the combined treatment of specific intensive physiotherapy and RSC brace achieved improved Cobb angel, better esthetic and the operation was avoided.

## Implications

Early detection could improve the results in all parameters, school screening or other systematic method for early detection is highly recommended

Specialized team work of scoliosis rehabilitation - Orthopedic physician, Orthotist, Physiotherapist, and compliant patient can achieve better result even in moderate-severe cases.

